# Motivation and Life Circumstances Affecting Living Habits Prior to Gastrointestinal Cancer Surgery- An Interpretative Phenomenological Analysis

**DOI:** 10.1177/00469580231170544

**Published:** 2023-05-26

**Authors:** Karin Blomquist, Carina Wennerholm, Carina Berterö, Per Sandström, Bergthor Björnsson, Jenny Drott

**Affiliations:** 1 Division of Nursing Science, Department of Health, Medicine and Caring Sciences, Linköping University, Linköping, Sweden; 2 Department of Surgery, Department of Biomedicine and Clinical Sciences, County Council of Östergötland, Linköping University, Linköping, Sweden

**Keywords:** habits, mental health, motivation, neoplasm, nursing

## Abstract

The aim was to explore the patient’s experiences to get insights into their living habits prior to gastrointestinal cancer surgery. An interpretative phenomenological analysis (IPA) approach was used. Six in-depth interviews with participants recruited from a hospital in southeast Sweden. The IPA analysis identified 3 themes: *The influence of the cancer diagnosis on awareness and motivation, Life circumstances affecting living habits*, and *Activities bringing mental strength*. The participants expressed their motivation level and circumstances in life. Various types of activities and support promoted physical and mental health. Motivation level and circumstances in life both influence living habits. Various kinds of activities and support promote patients’ physical and mental health. Nurses need to investigate patients’ experiences when developing person-centered support to achieve health-promoting behavior prior to cancer surgery.

**What do we already know about this topic?**
Prehabilitation aims to support improved living habits, support patients in building their mental strength prior to surgery and facilitate postoperative recovery.
**How does your research contribute to the field?**
Attention should be given to patients’ living habits early in the preoperative oncological treatment phase, and patients’ mental health and motivation should be promoted.
**What are your research’s implications toward theory, practice, or policy?**
Diverse types of activities and support promote patients’ physical and mental health. Nurses need to investigate patients’ experiences when developing person-centered support to achieve health-promoting behavior prior to cancer surgery.

## Introduction

Patients with cancer scheduled for surgery require a high functional capacity to benefit from the treatment, and patients’ living habits are important. Frail patients have reduced physiological reserves and increased risks of postoperative complications that prolong hospital time, increase readmission incidence and decrease survival.^1^ Prehabilitation to support improved living habits supports patients in building their strength prior to surgery and may facilitate postoperative recovery.^2^ Prehabilitation programs include education and therapeutic exercises designed to meet the patients’ functional, psychological, and social needs. Previous research has explored the impact of physical training, nutritional care, psychological counseling, and preoperative smoking cessation.^1,3^

There is a growing body of research on preoperative multimodal optimization programs (prehabilitation) for patients before gastrointestinal cancer surgery.^4,5^ Exercise, nutrition, anxiety reduction and/or smoking cessation have been shown to improve physical recovery after gastrointestinal cancer surgery and significantly decrease the length of hospitalization.^4-6^ In major gastrointestinal cancer surgery, studies have shown that postoperative functional capacity benefits from Prehabilitation. However, there are still questions about what level of impact multimodal programs have on postoperative complications and health-related quality of life (HRQoL).^2^ Multimodal prehabilitation may reduce morbidity after abdominal surgery, but research about complications and mortality is sparse.^4,7^

Most of the literature evaluating patient adherence to prehabilitation programs has shown the need to make the programs patient-centered by taking personal thoughts, conditions, and practical barriers and facilitators should be considered.^8-10^ Patients with depressive symptoms have been shown some benefit from prehabilitation interventions.^11^ However, there is insufficient evidence to confirm which preoperative psychological interventions are most effective.^12^ Individualized optimization strategies in prehabilitation programs should focus on different stressors already present in the preoperative phase and may be able to influence the patient’s recovery and reduce postoperative complications.^9,13^ By proactively identifying patients at risk for deterioration and applying the correct set of strategies (eg, education and support) at the appropriate time, superior outcomes may be achieved.^14,15^ Cancer patients scheduled for surgery are a vulnerable group. Responsibility for the nurses and health care professionals who take care of people in situations where they face vulnerability is thus to support the exposed persons in attaining improved health. To be able to do this optimally, an in-depth understanding of the patients’ experiences and views is necessary. The Health Promotion Model (HPM) integrates several constructs to improve health and living habits. Self-efficacy is a central construct of the HPM.^16^ Professionals, such as nurses and physicians, are responsible for facilitating their patients’ perceived control, empowerment, and resilience. HPM encourages healthcare professionals to provide positive resources to support patients in achieving specific behavioral changes.

There is a lack of qualitative evidence on living habits in cancer care, and there is a lack of in-depth understanding of the patients’ views and experiences of physical training, nutritional care, psychological support, and smoking/alcohol cessation in the preoperative phase (prehabilitation components). Therefore, patients’ views and influences regarding prehabilitation components may understand their point of view and help direct care better or differentiate between features to aid in tailoring interventions. The aim was to explore the patient’s experiences to get insights into their living habits prior to gastrointestinal cancer surgery.

## Methods

### Design

The study employed a qualitative research approach with in-depth interviews. An interpretative phenomenological analysis (IPA) approach was used. The study recruited and included patients from a university hospital. The COnsolidated criteria for REporting Qualitative research (COREQ) Checklist were used to ensure quality in this study.^17^

### Participants, Sampling, and Data Collection

The inclusion criteria were adult patients (at least 18 years of age) receiving preoperative oncological treatment and scheduled for abdominal cancer surgery and the capacity to give written informed consent. The exclusion criteria were as follows: patients with cognitive dysfunction and patients who did not speak and understand Swedish language (communicate fluently) and were unable to go through the normal consenting process (in line with Good Clinical Practice/GCP guidelines). The sampling strategy was purposive and convenient and aimed at achieving variation in sex, age, and cancer diagnosis/treatments. The included patients in this study could be considered representative of patients with gastrointestinal cancer. A GCP-competent clinical nurse recruited the patients in surgical care and informed them about the study orally and in writing. Patients received an information letter containing a description of the purpose of the study, an informed consent form and a postage-paid return envelope. Patients who consented to participate were contacted by telephone by the interviewer. In total, 6 participants were interviewed between November 2020 and March 2021. Patient characteristics are presented in Table 1. Pseudonyms were assigned to the participants so that anonymity of participants was safeguarded.

**Table 1.** Patient Characteristics (With Pseudonyms).

Pseudonyms of participants	Age	Sex (female/male)	Marital status	Diagnosis, malignancy	Oncological treatment
Ali	56	M	Single	Gastric cancer	Chemotherapy
Marianne	68	F	Married	Esophageal cancer	Radio-/chemotherapy
Ralf	78	M	Married	Rectal cancer	Radiotherapy
Sarah	51	F	Married	Esophageal cancer	Radio-/chemotherapy
Thomas	76	M	Married	Rectal cancer	Radio-/chemotherapy
Viola	77	F	Married	Rectal cancer	Radio-/chemotherapy

The research group developed an interview guide including open-ended questions (see Table 2). The interview questions were based on the specific study purpose and influenced by relevant literature/important topics in living habits. The guide underwent pilot testing in one patient to ensure clarity; no adjustments were made, and the interview was included in the analysis. The interviews were performed as a conversation, within these areas. Probing and looping questions were used continuously during the interviews in relation to the patients’ specific views and experiences.

**Table 2.** Interview Guide.

(a)	Please, tell me about your living habits, how you have lived your life until today—(Your perspectives and habits regarding exercise, nutrition, smoking/alcohol, and mental health).
(b)	Please, tell me about your thoughts about your mental health and how it can affect your living habits?
(c)	Please, tell me about your thoughts about how your living habits can influence the surgical procedure you are planned for and the effect on your recovery after surgery?

The interviews were conducted in digital video meetings (owing to COVID-19 safety measures) and were digitally recorded. The first author, a female clinical nurse specialist in surgical care with long experience in cancer care, conducted the interviews. The interviews were held 4 to 17 days (median 10 days) prior to surgery. The interviews lasted between 23 and 64 min (median 55 min).

### Data Analysis

The first author transcribed the interviews verbatim; the data were analyzed with interpretative phenomenological analysis (IPA) to investigate how individuals make sense of their lived experiences, mainly during life changes.^18,19^ The analysis was guided by the 6-step IPA process described by Smith et al^19^ For immersion in the participants’ narratives, the recorded interviews and transcribed text were listened to, read, and re-read to absorb the essence of their lived experiences (Step 1). A detailed set of initial notes was made with a descriptive core, linguistic and conceptual notes, and initial interpretative comments (Step 2). The comprehensive notes that reflect the source material were transformed into emerging themes (Step 3). All transcripts were separately analyzed; the next step was designed to search for patterns across cases (Step 4). A reconfiguring and relabeling process began, and the themes were identified (Step 5). Data coding, coding tree, and identifying themes were derived from the data (Step 6). Data collection of IPA research aims to get rich and thick descriptions of the participants’ lived experiences.

### Rigor

To ensure credibility in IPA analysis, sensitivity to context, commitment, and rigor in undertaking the analysis. The research group continuously scrutinized the analysis. To illuminate the findings and exemplify the interpretive text, the participants’ words are quoted extensively, integrated with the text and inserted as block quotes. The interviews were richly detailed, and the study aim and validity were achieved, consistent with the principles of Yardley.^19,20^

### Ethical Considerations

This study was approved by the Research Ethics Committee in Sweden (No. 2020-04289). Oral and written informed consent was obtained in compliance with the Declaration of Helsinki.^21^

## Results

Three themes were identified in the interpretative analysis: (i) *The influence of the cancer diagnosis on awareness and motivation*, (ii) *Life circumstances affecting living habits*, and (iii) *Activities bringing mental strength*. These themes represent the essence of the participants’ lived lifeworld according to their living habits during cancer prior to surgery.

### Theme 1: The Influence of the Cancer Diagnosis on Awareness and Motivation

The participants expressed their thoughts and feelings in the period shortly after the cancer diagnosis. Existential emotions such as *“chaos”* and *“anxiety”* appeared and made them aware of their rapidly changing life situation. These feelings were experienced as a normal reaction to the crisis and life-changing bad news. The participants attempted to be in the present, focusing on living. There was a process of accepting the cancer diagnosis and becoming able to live with cancer and its treatments for a relatively long period of life.

*“This anxiety that you had in the beginning there. . .. there was a way to realise where you are.” –* Thomas

This awareness of life-threatening cancer awakened feelings of uncertainty. There was uncertainty due to a lack of knowledge about cancer and how it would affect life, and patients thought about how they could maintain an ordinary life. This awareness also brought uncertainty regarding the future and negative information about the prognosis. The participants reflected on being forced to live with uncertainty for the rest of their lives.

*“You live, so to speak, in a bubble of uncertainty . . . where is this taking me?”*

– Thomas

*“This is like. . ..no one has really sat down with me and explained to me . . . what I could do to make it . . . become good for me.” –* Sarah

Some participants expressed the sentiment, *“Finally!”* when diagnosed with cancer. The uncertainty and the feeling that something was wrong were verified. The body and mind signal it with various symptoms. When the cancer was confirmed, the patients’ confidence grew, and they felt convinced that they would manage the disease. Living day by day and focusing on taking care of themselves contributed to a better outcome,

*“It’s not like handing in the car for service. . . you need to contribute by yourself.. . . I think that’s really important” –* Marianne

Participants were mentally preparing for major surgery and reflecting upon this issue with someone outside their nearest social network. They were aware of the major surgical procedure, which would place heavy individual demands on them. Issues regarding mental and physical support, aid, and what to eat and drink were raised. The participants were aware of the effects of these factors on their lives and wanted to be prepared and motivated to oversee their recovery and living habits.

*“if the surgery and everything goes well, there won’t be a change in life, the difference is that you might have a stoma” –* Ralf

Awareness of the situation exists, and motivation was a factor that could affect their lives during cancer, leading to a more inactive everyday life than the participants wanted and were accustomed to. Some participants saw themselves as active persons with many activities and hobbies but became inactive. They felt that it was meaningless to do these activities.

*“It’s hard every day when getting out of bed . . . finding something that feels meaningful” –* Viola

There could also be thoughts such as *“to what use,”* questioning why there should be exercise and sound performance as before. Despite these thoughts, the living habits that the participants had developed over many years were the essence of the maintenance of health-related behavior despite a lack of motivation.

### Theme 2: Life Circumstances Affecting Living Habits

The participants described earlier experiences in life and how these might affect living habits. Some participants had heart disease and became aware that life is fragile and requires self-care. Others struggled with depression and symptoms such as fatigue and severe difficulties with memory. These circumstances affected their ability to maintain their living habits. Some participants reported diverse types of skeletal injuries leading to impaired mobility and pain, which limited physical activity. Long-term effects led to acceptance and adjustment of the situation, and their living habits were not negatively impacted in the long term. They were as active as they wished to be.

*“I have pain . . . it would never stop me . . . and I don’t take support from anyone else because I’m feeling pain . . . there is always something positive in the end.” –* Viola

Some participants mentioned prior surgical experiences and referred to them when talking about recovery. They recalled positive experiences of their ability to recover and embraced self-efficacy for their planned surgical procedures. Despite their confidence in an excellent recovery, the older participants reflected upon the fact that they were older now.

One participant had acute abdominal surgery a few months prior and had the new positive experience that being physically active after surgery, even when feeling weak, led to a quick recovery. Previous experiences will be used as a force to manage this situation and thoughts of healing. Being empowered will bring the strength to manage new circumstances, such as oncological treatment. Participants who know what happens and why and are confident in their ability to manage will develop their living habits by adjusting their diet and physical activity to prepare for major surgery.

*“. . . best possible result. . .. I’m sure. . .. I have really experienced that after this acute operation.” –* Marianne

Participants had experiences with changing their living habits earlier in life. The causes were sometimes medical conditions and concerns about weight gain. In particular, patients focused on their eating and smoking habits. Some participants had diabetes, and when informed that changing their eating habits would be beneficial for their health, they rapidly changed their eating habits and made plans to continue with these new habits. Smoking cessation was another change that some patients made after having a myocardial infarction: *“this is a date you never forget”.*

*“The only thing I could influence then was my living habits.”* – Ralf

There were also counseling experiences for eating habits due to being overweight caused by immobility. The dietary recommendations patients received when diagnosed with cancer became confusing and were challenging to manage because they were directly contrary to the previous recommendations.

*“* . . . *but yes, I’m scared . . . think if I only continue to eat like this afterwards? . . . then I have thoughts of. . . I will roll forward and become really fat (laughs)*” *–* Sarah

The participants expressed similar thoughts about food and healthy eating and drinking habits. The use of alcohol was presented as a habit that was unprioritised and unrewarding during cancer. Older participants expressed that they could have eaten more vegetables earlier in life: *“We have tried to add more vegetables over time . . . but we should have done that much sooner.”* Their circumstances in life were different then, and lifestyle issues were not in focus at that time. Patients had thoughts of what could have caused their cancer.

### Theme 3: Activities Bringing Mental Strength

The participants expressed that those activities, such as physical/social activities and support from health care professionals, raised their physical and mental energy levels. Participants identified themselves as active persons, but there were differences in their definitions of physical activity. Activity levels among participants were variable, such as exercising every day at the gym, walking several hours in the forest, taking the dog for a walk, or being engaged in a sports association. These are all ordinary physical activities, and the participants saw themselves as active and considered it a positive quality.

The participants related physical strength to positive feelings. They expressed that it was easier to remain in a good mood when they had physical strength or at least regained their power before the next dose of chemotherapy. “*It’s so nice. . . this feeling of regaining strength.”* Patients noted that physical activity strengthened the body and mind, and even more favorably, they found that it enhanced their adjustment to the situation; for example, one participant expressed that sentiment as follows:

*“I feel I haven’t the same strength in the body . . . but strangely, when I thought, ‘Now I will go to the gym,’ it disappears . . . unbelievable . . . the pain just disappears because I think of something else . . . everything is inside here (pointing to his head).” –* Ali

On the other hand, physical weakness elicited adverse feelings: patients did not recognize themselves and feared that their condition would worsen: *“You don’t feel like yourself”.* Physical energy levels affect the ability to maintain prior behavior. Physical symptoms caused by cancer itself or by the side effects of oncological treatment were common. Support from healthcare professionals was crucial. The participants who felt that they received insufficient support experienced difficulties managing their living habits, particularly eating and physical activity, in an unsatisfying manner. Loneliness sometimes occurs, negatively affecting the patients’ mental strength. Receiving support too late made the participants frustrated.

*“After that, there haven’t been any problems . . . I know exactly what I shall do . . . but I had not a clue before . . . so that was like super hard.” –* Sarah

*“I have a consulting nurse, and I think it has been functional if I need anything, but in a way I miss a steady hand that follows me and knows how I feel on a weekly basis . . . the same person.” –* Thomas

The participants highlighted their social interactions with family, friends, and other persons in their environment as an essential part of life. Participants experienced social activities, including support from the nearest members of their social network, as strengthening their health. They identified their life partner as their most robust mental support; if needed, the partner rendered practical assistance in everyday life with practical support. Feelings of losing their habitual role in their social context appeared. The experience of maintaining their traditional role and identity in their social lifeworld brought significant mental strength during cancer.

## Discussion

The main findings show that the participants maintained and improved their living habits before cancer surgery. The importance of preserving their identity and their habitual roles during the cancer continuum to strengthen their mental health was a common element. Findings postulated that diverse types of support facilitated the maintenance or development of presurgical living habits. This illustrates that health promotion has a crucial role in cancer care. There is a growing body of research on preoperative multimodal optimization programs (prehabilitation) for patients before gastrointestinal cancer surgery.^4,5^ Although the programs include interventions, they do not always motivate patients to behavioral and actual changes.^1^ In the HPM, the outcome is a health-promoting behavior to enhance functional ability and health.^16^ The most consistent predictors of health behavior are self-efficacy, how barriers are perceived and social support. A high level of self-efficacy leads to a reduced perception of barriers.

The findings illustrate the participants’ awareness of life and death and how they struggled to find meaning in their everyday life. Motivational factors that motivated them earlier in life sometimes weakened, and they appeared to need a change in their source of motivation to maintain previous habits. Participants’ awareness of the situation can influence optimizing conditions for surgery and can be a motivational factor. In the HPM, the consequences of actions are an influential factor in motivating behavioral changes. Positive results lead to feelings of benefit.^16^ Participants who see benefits from their maintenance of living habits are likely to gain an increased sense of self-efficacy, reducing their perceived barriers. They are more likely to commit to optimizing their body and mind for cancer surgery. If negative consequences appear when cancer symptoms and side effects of treatment are present, there are difficulties in determining what health-related living habits can improve in their situation and maybe also difficulties in choosing the most effective interventions. The symptom burden and side effects of treatment are strong predictors of failure to perform physical exercise.^22^ The participants expressed how circumstances in life had affected them and whether their previous life experiences facilitated or burdened their living habits when a particularly demanding event appeared. In the HPM, a history of health-related behavior strengthens habits and is a key factor in consolidating behavior.^16^ The participants illustrate this through the maintenance of, for example, regular physical activity and a nutritious, well-cooked diet despite the circumstances. Patients scheduled for cancer surgery may perceive the preoperative phase as a good opportunity to change alcohol and smoking habits. A non-judgmental style of support is recommended, and findings from a qualitative study show that nurses and health professionals should provide supportive care congruent with the patient’s preferences and needs.^23,24^ The study also highlights the importance of active and bidirectional communication with patients, and interprofessional collaboration may optimize patient outcomes and the use of resources.^25^ Activities in the participants’ lives elevated their mental energy levels, and the findings illustrated that insufficient activity in certain areas could result in decreased mental strength. Social activities and support were prominent in ensuring that the participants retained their mental power. The HPM posits that interpersonal influences, including family, friends and healthcare staff are predictors of the maintenance of health-related behavior.^16^ The participants highlighted their social interactions with family, friends, and other persons in their environment as an essential part of life. Participants experienced social relationships, including support from the nearest members of their social network, as strengthening their health; this is in line with existing literature.^26^ HRQoL and mental health have shown significant improvement preoperatively with exercise.^27^ Findings from studies in patients with cancer suggest that everyday life activities and habits define and construct agency and that these constructions are tightly linked to the person’s overall life situation, physical abilities, and cultural context. Therefore, a holistic approach is essential to improve patients’ health and well-being.^28,29^ This agrees with our study and strengthens the importance of our study design and to study patients’ in-depth perspective of living habits prior to gastrointestinal cancer surgery.

### Limitations and Strengths

This study had a small sample size and a short enrollment period, and recruitment was limited by other studies being conducted in parallel. However, the idiographic nature of IPA often necessitates and validates using a small, homogenous sample.^18,19^ Smith et al^19^ highlight that the sample size is contextual and must be considered based on the specific study. The included patients in this study could be considered representative of patients with gastrointestinal cancer. The pandemic situation might have negatively affected the data collection (by forcing the use of digital rather than in-person meetings), but digital interviews complement face-to-face interviews. The interviews were richly detailed, and the study aim and validity were achieved, consistent with the principles of Yardley.^19,20^ The research team is experienced in cancer care, facilitating awareness and dedication in the interview situation and promoting context sensitivity, commitment, and rigor in the analysis. The participants in this study expressed that activities, such as physical/social activities and support from nurses and health care professionals, raised their physical and mental energy levels.

## Clinical Implications

The results from our study encourage the possible clinical application of a system in which healthcare professionals provide person-centered supportive cancer care to achieve specific health-promoting activities. Clinical interventions of prehabilitation programs and the included components will be performed in future research. Future research about implications from the study’s findings are that healthcare professionals facilitate patients’ empowerment processes to individualized optimization strategies in prehabilitation programs to provide positive resources to support patients in achieving and sustain specific behavioral changes. The findings highlight areas where support from nurses and healthcare professionals may be crucial. The participants who received insufficient support experienced difficulties with their living habits, particularly eating and physical activity. The important findings from our study encourage the possible clinical application of a system in which healthcare professionals provide person-centered supportive cancer care to achieve specific health-promoting activities. Attention should be given to patients living habits already early in the preoperative oncological treatment phase.

## Conclusion

Motivation level and circumstances in life both influence living habits. Diverse types of activities and support promote patients’ physical and mental health. Nurses need to investigate patients’ experiences when developing person-centered support to achieve health-promoting behavior prior to cancer surgery. Nurses have an obligation to communicate the importance of living habits to support patients with cancer prior to surgery. Living habits might be a sensitive topic, but on the other hand, the findings of this study advanced the current understanding to facilitate the development of a person-centered supportive health-promotion approach aimed at improving health.
